# Syntheses and crystal structure of a (2,6-diiso­propyldi­naphtho­[2,1-*d*:1′,2′-*f*][1,3]dithiepin-4-yl)(phen­yl)methanol atropisomer

**DOI:** 10.1107/S2056989023001706

**Published:** 2023-02-28

**Authors:** Neil Beare, Gavin F. Painter, C. John McAdam

**Affiliations:** a BDG Synthesis, PO Box 38627, Wellington Mail Centre 5045, Wellington, New Zealand; b Ferrier Research Institute, Victoria University of Wellington, PO Box 33436, Lower Hutt 5046, New Zealand; c Department of Chemistry, University of Otago, PO Box 56, Dunedin 9054, New Zealand; University of Aberdeen, United Kingdom

**Keywords:** crystal structure, atropisomer, bi­naphthalene di­thiol, asymmetric synthesis, hydrogen bonds, C—H⋯π contacts

## Abstract

The racemic title compound, C_34_H_32_OS_2_, comprises an atropisomeric binaphthyl di­thio­acetal substituted at the methyl­ene carbon atom with a chiral benzyl alcohol. The two naphthalene ring systems are additionally substituted at the 3,3′-position with isopropyl groups. The overall stereochemistry is defined as *aS,R* and *aR,S*. The hydroxyl group forms an intra­molecular O—H⋯S hydrogen bond to one of the sulfur atoms. The crystal structure contains weak C—H⋯π inter­actions that link the mol­ecules into extended arrays.

## Abstract 2

The mol­ecular and crystal structure is reported for the major diastereoisomer formed in a sterically hindered reaction of a substituted di­naphtho­dithiepine. The structure lends support to a reaction mechanism and transition state postulated to explain the experimentally determined preferential formation of this diastereoisomer over the minor component.

## 1. Chemical context

In the continuing pursuit of stereoselective synthetic methodology, steric considerations play an important role. Indeed, defined by steric limitations are the atropisomers of biaryl compounds formed as a result of restricted rotation about the connecting single bonds (Cen *et al.*, 2022 ▸; Wencel-Delord *et al.*, 2015 ▸; Cheng *et al.*, 2021 ▸). For 1,1′-bi­naphthalenes, functionalization of the 2,2′ positions with a di­thia­pine ring helps to lock the atropisomers against inversion and facilitates studies of diastereoselective reactions. An example of such reactions is the attack of the sulfur-stabilized di­naphtho­dithiepine carb­anion on a prochiral electrophile Delogu *et al.*, 1991 ▸; Beare *et al.*, 2023 ▸). Reaction of the organolithium of di­naphtho­[2,1-d:1′,2′-*f*][1,3]dithiepine, and various substituted derivatives with benzaldehyde (and other prochiral ketones) proceeded in high chemical yield and gave readily separable alcohol products, allowing the diastereomeric excess to be qu­anti­fied (Pa­inter, 1995 ▸; Beare, 1999 ▸). The results suggested the structure of the organolithium species is significant in determining the stereoselectivity, and that in all cases the same diastereoisomer (*aS,R*/*aR,S*) forms the major product (Delogu *et al.*, 1991 ▸; Beare *et al.*, 2023 ▸).

**Figure 1:**
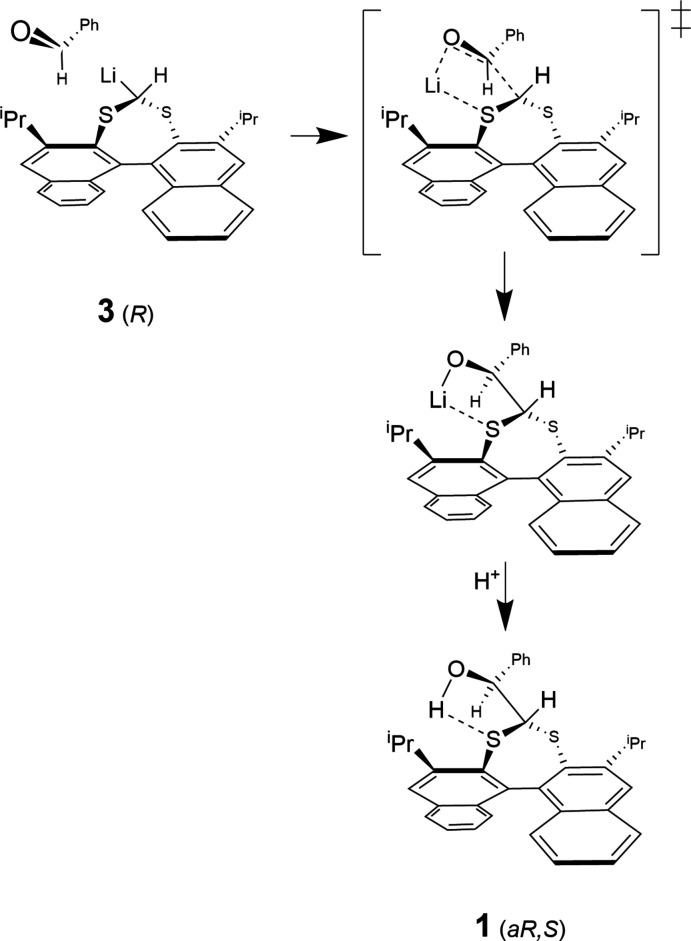
Proposed reaction and transition state for the carbanion attack of the *R* atropisomer at the *Re* face of benzaldehyde.

This work reports the synthesis and single-crystal X-ray structure of the major diastereoisomer of (2,6-diiso­propyldi­naphtho­[2,1-*d*:1′,2′-*f*][1,3]dithiepin-4-yl)(phen­yl)methanol, C_34_H_32_OS_2_, **1**, formed from the reaction of the carbanion of 2,6-diiso­propyldi­naphtho­[2,1-*d*:1′,2′-*f*][1,3]dithiepine (**2**) with benzaldehyde. The stereochemistry is confirmed as *aS,R*/*aR,S*. We postulate that the preference for this geometry is a transition state that minimizes steric inter­actions between the incoming ketone and proximal 3,3′ bi­naphthalene substituents, isopropyl groups in the case of **1**. Intra­molecular O—H⋯S hydrogen bonds (described below) also provide a model for predicted lithium–sulfur inter­actions that stabilize the transition state and li­thio salt, prior to the quenching of the reaction. A reaction mechanism showing carbanion (**3**) attack of the *R* atropisomer at the *Re* face of benzaldehyde to form the major *aR*,*S* diastereoisomer is illustrated in Fig. 1 ▸. 
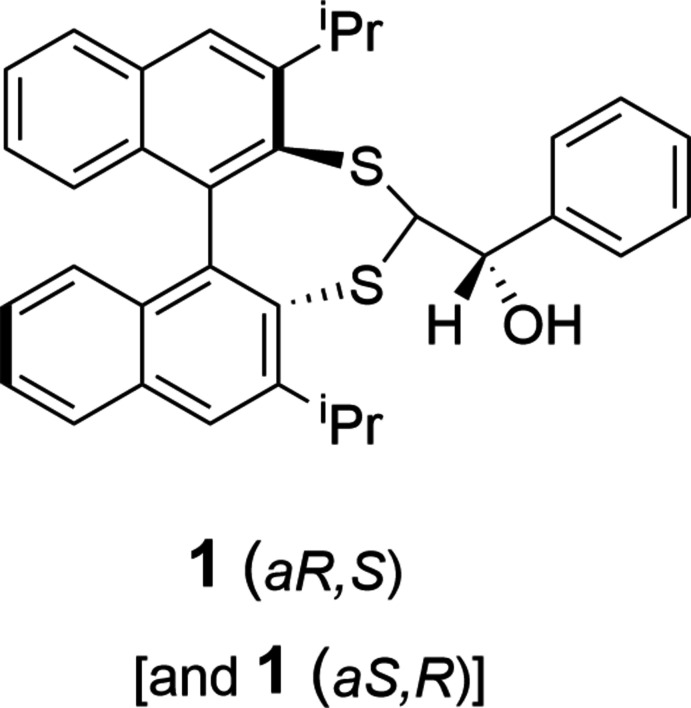


## 2. Structural commentary

**Figure 2:**
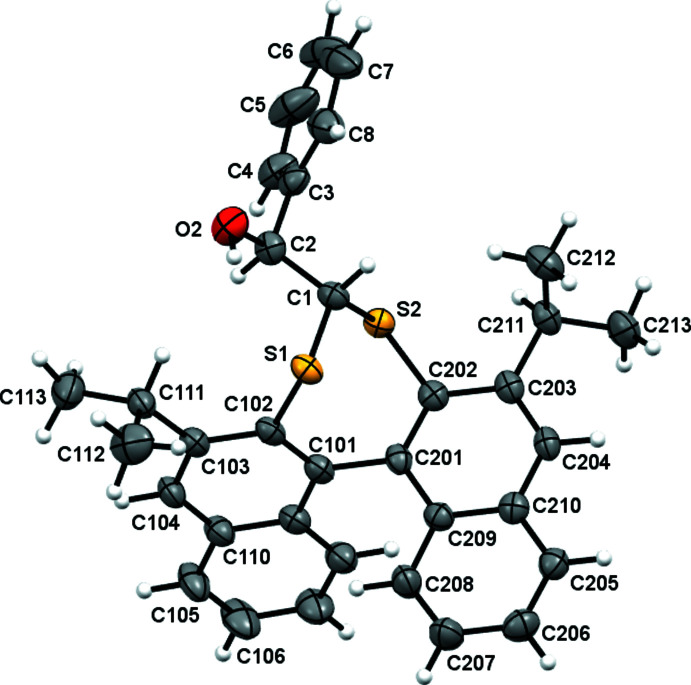
The mol­ecular structure of **1** with displacement ellipsoids drawn at the 50% probability level. Carbon atoms C107–C109 follow the logical progression but their labels are omitted for clarity.

**Figure 3:**
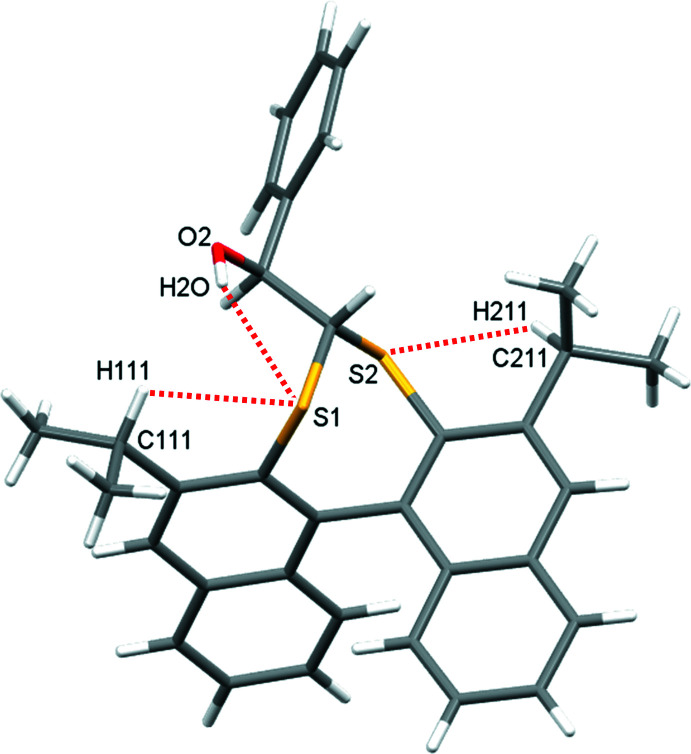
The intra­molecular O—H⋯S hydrogen bond and short C—H⋯S contacts of **1**.

In **1** (Fig. 2 ▸), a 1,1′-linked bi­naphthalene is functionalized at the 2,2′ positions with disulfaneyl­methane. The seven-membered ring formed locks the bi­naphthalene ring system into *R* and *S* atropisomers of pseudo-*C_2_* symmetry. The individual naphthalene ring systems are predictably flat, with r.m.s. deviations from the ten-atom mean plane of 0.017 and 0.026 Å for C101–C110 and C201–C210, respectively. The C102—C101—C201—C202 torsion angle is −68.8 (4)°, and the dihedral angle between the naphthalene ring mean planes is 70.4 (1)°. The structure is extended with a chiral benzyl alcohol substituent on the methyl­ene bridge carbon atom, giving *aS,R* and *aR,S* enanti­omer pairs. The alcohol group of the mol­ecule is positioned such that an intra­molecular hydrogen bond forms to one of the bridge sulfur atoms (Table 1 ▸, Fig. 3 ▸). The same feature has been observed in the closely related structure with Cambridge Structural Database refcode NEWVOE (Beare *et al.*, 2023 ▸). Completing the structural description are isopropyl residues on the 3- and 3′-positions of the bi­naphthalene unit that are arranged so as to minimize steric inter­action with the thio­acetal core, but result in short contacts between the methane­triyl hydrogen atoms and adjacent sulfur atoms of the seven-membered ring (H⋯S = 2.72–2.73 Å, Fig. 3 ▸).

## 3. Supra­molecular features

**Figure 4:**
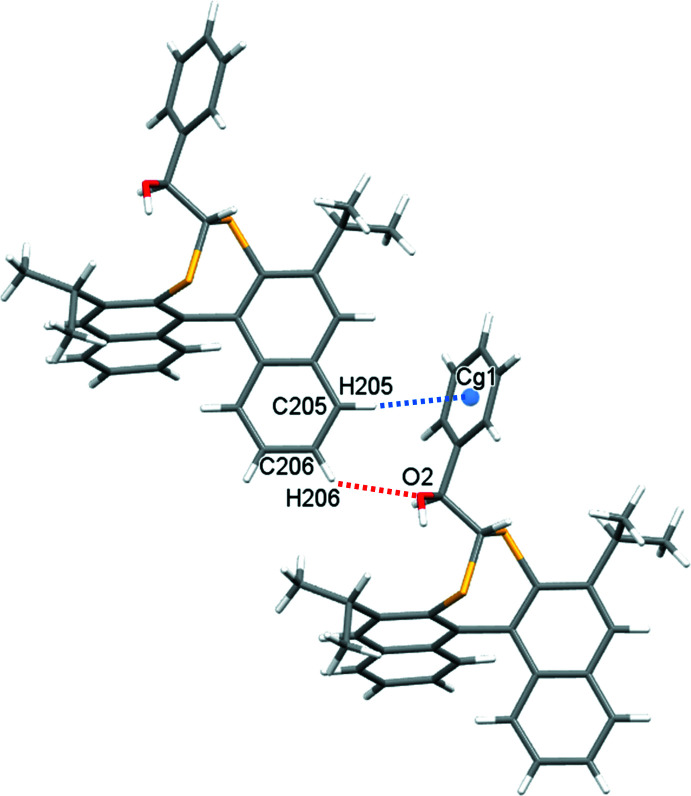
C206—H206⋯O2 (red dashed lines) supported by weak C205—H205⋯π(*Cg*1^i^) inter­actions (dashed blue lines), which generate chains of **1** propagating in the *a*-axis direction. *Cg*1 is the C3–C8 ring centroid; symmetry code: (i) 1 + *x*, *y*, *z*.

**Figure 5:**
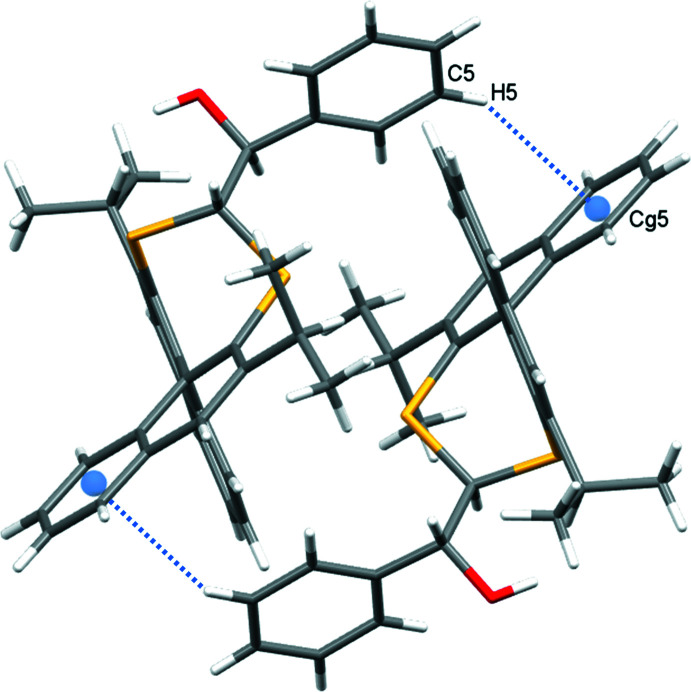
Pairwise C5—H5⋯π(*Cg*5^ii^) inter­actions in **1**, which generate inversion dimers *Cg*5 is the C205–C210 ring centroid; symmetry code: (ii) 1 − *x*, 1 − *y*, 1 − *z*.

In the crystal of **1**, a weak C—H⋯O hydrogen bond (Veljković *et al.*, 2011 ▸) between naphthalene atom H206 and the adjacent alcohol oxygen atom generates chains propagating in the *a*-axis direction (Table 1 ▸, Fig. 4 ▸). The motif is supported by a weak Malone Type II C—H⋯π contact (Malone *et al.*, 1997 ▸) between a naphthalene hydrogen atom and the benzyl aromatic ring (H205⋯*Cg*1 = 3.01 Å; *Cg*1 is the centroid of the C3–C8 ring). A further Malone Type III C—H⋯π inter­action (H5⋯*Cg*5 = 3.03 Å; *Cg*5 is the centroid of the C205–C210 ring) forms inversion-related dimers (Table 1 ▸, Fig. 5 ▸).

## 4. Database survey

A search of the Cambridge Structural Database (Groom *et al.*, 2016 ▸) gives only four hits for the di­naphtho­dithiepine fragment, including a di­naphtho­dithiepine *S*-oxide (refcode JITTEL; Delogu *et al.*, 1991 ▸) and TEVQUK and closely related NEWVIY and NEWVOE from this research group (Beare & McAdam, 2023 ▸; Beare *et al.*, 2023 ▸). The 1,1′-bi­naphthalene framework with 3,3′ isopropyl groups is unprecedented.

## 5. Synthesis and crystallization

**Figure 6:**
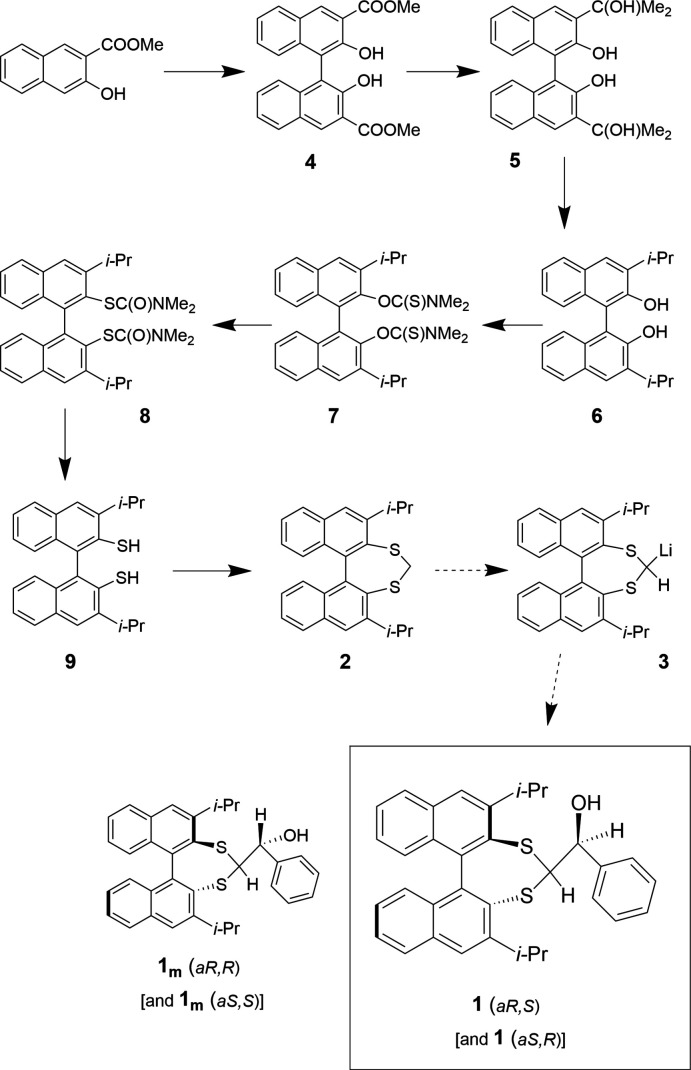
Preparation of **1**.

The synthesis of **1** is a multistep process (Fig. 6 ▸), but can be summarized as follows: preparation of the isopropyl-substituted bi­naphthalene diol (**6**); conversion to the di­thiol (**9**) exploiting the Newman–Kwart thermorearrangement of the bis-*O*-thio­carbamate ester (Kwart & Evans, 1966 ▸; Newman & Karnes, 1966 ▸); Lewis acid-catalysed thio­acyl­ation to form the seven-membered 1,3-dithiepine ring (**2**); and finally reaction of the sulfur-stabilized carbanion with the prochiral electrophile benzaldehyde.

*3,3′-Diisopropyl-[1,1′-bi­naphthalene]-2,2′-diol* (**6**): a previously reported synthesis of precursor diol **5** was in low overall yield due to an inefficient oxidative dimerization of 3-hy­droxy-2-naphthoic acid (Cram *et al.*, 1978 ▸). In this work we utilized the effective catalytic oxidation of methyl 3-hy­droxy-2-naphtho­ate to prepare diesterdiol **4** (Noji *et al.*, 1994 ▸). Returning to Cram’s procedure, treatment of **4** with MeLi produced **5**. Hydrogeno­lysis of this using tri­ethyl­silane and gaseous boron trifluoride (Fry *et al.*, 1978 ▸) gave the diiso­propyl­diol **6** as a white solid, m.p. 453–454 K (81%): ^1^H NMR (500 MHz) δ (ppm): 1.40 and 1.41 [2 × (6H, *d*, Me)], 3.49 (2H, *sept*, C*H*Me_2_), 5.18 (2H, OH), 7.07 (2H, *d*, binap H5,5′), 7.24 (2H, *ddd*, binap H7,7′), 7.35 (2H, *ddd*, binap H6,6′), 7.85 (2H, *s*, binap H4,4′), 7.86 (2H, *d*, binap H8,8′). ^13^C NMR (50 MHz) δ (ppm): 22.7 (Me), 28.0 (*C*HMe_2_), 110.9 (2*C*, binap *C*), 123.9, 124.0, 126.5, 126.7 & 128.0 (5 × 2*C*, binap *C*H), 129.6, 131.9, 137.4 & 151.5 (4 × 2*C*, binap *C*).

*3,3′-Diisopropyl-[1,1′-bi­naphthalene]-2,2′-di­thiol* (**9**): **6** was converted *via* the bis-*O*-(*N*,*N*-di­methyl­thio­carbamate) **7** to the bis-*S*-(*N*,*N*-di­methyl­thio­carbamate) **8**. NaH (2.5 equiv.) was added to a solution of **6** in DMF at 273 K. After 1 h, *N*,*N*-di­methyl­thio­carbamoyl chloride was added and the mix stirred at 358 K for 3 h. After cooling again to 273 K, the product was precipitated by addition of 5% KOH solution. Flash chromatography (SiO_2_/CH_2_Cl_2_) and recrystallization from toluene solution gave **7** as a white solid (82%). This was heated under N_2_ at 544 K for 30 min. Chromatography (SiO_2_/hexa­ne/CH_2_Cl_2_) and recrystallization from toluene solution gave the bis-*S*-thio­carbamate ester as a white solid, m.p. 486-487 K (83%). [**8**: ^1^H NMR (200 MHz) δ (ppm): 1.34 and 1.46 [2 × (6H, *d*, CH*Me*)], 2.51 (12H, *s*, NMe), 3.66 (2H, sept, C*H*Me_2_), 7.04 (2H, *d*, binap H5,5′), 7.11 (2H, *ddd*, binap H7,7′), 7.39 (2H, *ddd*, binap H6,6′), 7.84 (2H, *d*, binap H8,8′), 7.90 (2H, *s*, binap H4,4′). ^13^C NMR (50 MHz) δ (ppm): 23.4 and 24.8 (2 × CH*Me*), 31.5 *C*HMe_2_, 36.7 (NMe), 124.1, 125.1, 126.7, 127.2 and 127.8 (5 × 2*C*, binap *C*H), 128.0, 131.8, 134.2, 145.2 & 150.1 (5 × 2*C*, binap *C*), 166.1 (C=O)]. LiAlH_4_ (10 equiv.) was added to a suspension of **8** in THF at 273 K. The reaction mix was refluxed for 4 h, then cooled to 273 K, quenched (H_2_O) and acidified (conc. H_2_SO_4_). Extraction with Et_2_O was performed under an Ar atmosphere to avoid oxidation. Washing, drying (Na_2_SO_4_) and solvent removal *in vacuo* gave a yellow solid, which was purified by flash chroma­tography (SiO_2_/CH_2_Cl_2_) to give di­thiol **9**, m.p. 523–524 K (91%): ^1^H NMR (200 MHz) δ (ppm): 1.44 (12H, *d*, CH*Me*), 3.26 (2H, *s*, SH), 3.40 (2H, *sept*, C*H*Me_2_), 6.86 (2H, *d*, binap H5,5′), 7.16 (2H, *ddd*, binap H7,7′), 7.35 (2H, *ddd*, binap H6,6′), 7.82 (2H, *d*, binap H8,8′), 7.84 (2H, *s*, binap H4,4′). ^13^C NMR (50 MHz) δ (ppm): 23.3 and 23.4 (2 × CH*Me*), 31.9 *C*HMe_2_, 124.3, 125.1, 125.5, 126.6 & 127.9 (5 × 2*C*, binap *C*H), 131.0, 132.1, 132.6, 133.1 & 144.4 (5 × 2*C*, binap *C*).

*2,6-Diiso­propyldi­naphtho­[2,1-d:1′,2′-f][1,3]dithiepine* (**2**): to a solution of di­thiol **9** and di­meth­oxy­methane (1.05 equiv.) in CH_2_Cl_2_ under Ar at 273 K was added BF_3_·OEt_2_ (2.1 equiv.) dropwise. The reaction mix was allowed to warm to room temp. over 3 h, then stirred a further 12 h and quenched (H_2_O). The product was extracted with CH_2_Cl_2_, washed (5% KOH then water), dried and concentrated *in vacuo*. Chroma­tography on SiO_2_ (1:2 CH_2_Cl_2_:hexa­ne) and recrystallization from toluene solution gave **2** as a white solid, m.p. 536–537 K (95%): ^1^H NMR (300 MHz) δ (ppm): 1.40 and 1.46 [2 × (6H, *d*, CH*Me*)], 3.89 (2H, *sept*, C*H*Me_2_), 4.17 (2H, *s*, SCH_2_S), 6.93 (2H, *d*, binap H5,5′), 7.12 (2H, *ddd*, binap H7,7′), 7.42 (2H, *ddd*, binap H6,6′), 7.89 (2H, *d*, binap H8,8′), 7.90 (2H, *s*, binap H4,4′). ^13^C NMR (75 MHz) *d* (ppm): 23.8 and 24.7 (2 × CH*Me*), 31.6 *C*HMe_2_, 48.4 (SCH_2_S), 124.6, 125.5, 126.6, 127.4 and 127.9 (5 × 2*C*, binap *C*H), 129.2, 130.8, 134.1, 144.3 & 148.0 (5 × 2*C*, binap *C*).

*(2,6-Diiso­propyldi­naphtho­[2,1-d:1′,2′-f][1,3]dithiepin-4-yl)(phen­yl)methanol* (**1**): to a solution of di­thio­acetal **2** in THF under Ar at 173 K was added BuLi (1.4 *M* in hexa­nes, 1.3 equiv.) dropwise. The resultant deep-red solution stirred for 30 min confirms formation of the carbanion (**3**). Benzaldehyde (1.3 equiv.) was added dropwise and the mix stirred for a further 1 h at 173 K. The reaction was quenched (sat. NH_4_Cl), extracted with Et_2_O, washed (H_2_O), dried (MgSO_4_) and concentrated *in vacuo* gave the predicted mix of diastereo­isomers (78%, 69% d.e.). Chromatography on SiO_2_ (1:3 CH_2_Cl_2_:hexa­ne) eluted the major product first. Slow evaporation of an EtOH/H_2_O mix gave pale-yellow plates of **1** suitable for X-ray diffraction, m.p. 525–526 K: ^1^H NMR (200 MHz) δ (ppm): 1.26, 1.34, 1.35 and 1.61 [4 × (3H, *d*, CH*Me*)], 3.26 (1H, *s*, OH), 3.61 and 3.97 [2 × (1H, *sept*, C*H*Me_2_)], 4.27 (1H, *d*, C*H*OH), 4.82 (1H, *d*, SCHS), 6.88 and 6.94 [2 × (1H, *d*, Ar)], 7.08–7.17 (2H, *m*, Ar), 7.25–7.34 (5H, *m*, Ar), 7.37–7.50 (2H, *m*, Ar), 7.84 (1H, *s*, Ar), 7.86 and 7.93 [2 × (1H, *d*, Ar)], 7.97 (1H, *s*, Ar). ^13^C NMR (50 MHz) δ (ppm): 22.9, 24.0, 24.2 and 26.5 (4 × CH*Me*), 31.3 and 31.6 (2 × *C*HMe_2_), 75.2 (CH), 75.5 (CH), 124.6, 124.7, 125.5 and 125.7 (4 × Ar *C*H), 126.6 (Ar *C*), 126.7, 126.9, 127.1, 127.3, 127.5, 127.9, 128.4 and 128.7 (11 × Ar *C*H), 130.8, 131.0, 131.1, 134.1, 134.2, 139.4, 143.9, 144.7, 148.2 & 148.7 (10 × Ar *C*). Further elution gave the minor diastereoisomer pair **1_m_**, m.p. 527–528 K: ^1^H NMR (200 MHz) δ (ppm): 1.20, 1.34, 1.39 and 1.58 [4 × (3H, *d*, CH*Me*)], 3.28 (1H, *s*, OH), 3.80 and 3.96 [2 × (1H, *sept*, C*H*Me_2_)], 5.00 (1H, *d*, C*H*OH), 5.09 (1H, *d*, SCHS), 6.85 and 6.95 [2 × (1H, *d*, Ar)], 7.06–7.18 (2H, *m*, Ar), 7.26–7.49 (7H, *m*, Ar), 7.84–7.93 (4H, *m*, Ar). ^13^C NMR (50 MHz) δ (ppm): 22.3, 23.8, 24.7 and 26.8 (4 × CH*Me*), 31.3 and 31.4 (2 × *C*HMe_2_), 73.4 (CH), 78.6 (CH), 124.3, 124.7, 125.5, 125.6, 126.4, 126.6, 126.7, 127.4, 127.5, 127.9, 128.3 & 128.6 (15 × Ar *C*H), 129.3, 129.5, 130.7, 130.8, 130.9, 134.1, 141.5, 144.1, 144.2, 148.3 & 148.9 (11 × Ar *C*).

## 6. Refinement

Crystal data, data collection and structure refinement details are summarized in Table 2 ▸. All H atoms were refined using a riding model with *d*(C—H) = 0.95 Å, *U*_iso_ = 1.2*U*_eq_(C) for aromatic H, 1.00 Å, *U*_iso_ = 1.2*U*_eq_(C) for CH, 0.98 Å, *U*_iso_ = 1.5*U*_eq_(C) for methyl H atoms and *d*(O—H) = 0.84 Å, *U*_iso_ = 1.5*U*_eq_(O) for OH.

## Supplementary Material

Crystal structure: contains datablock(s) I. DOI: 10.1107/S2056989023001706/hb8055sup1.cif

Structure factors: contains datablock(s) I. DOI: 10.1107/S2056989023001706/hb8055Isup2.hkl

CCDC reference: 2244275

Additional supporting information:  crystallographic information; 3D view; checkCIF report

## Figures and Tables

**Table 1:** Hydrogen-bond and C—H⋯π geometry (Å, °) *Cg*1 and *Cg*5 are the centroids of the C3–C8 and C205–C210 rings, respectively.

*D*—H⋯*A*	*D*—H	H⋯*A*	*D*⋯*A*	*D*—H⋯*A*
O2—H2*O*⋯S1	0.84	2.53	3.069 (3)	123
C111—H111⋯S1	1.00	2.73	3.197 (3)	109
C211—H211⋯S2	1.00	2.72	3.163 (3)	107
C206—H206⋯O2^i^	0.95	2.58	3.284 (4)	132
C205—H205⋯*Cg*1^i^	0.95	3.01	3.886 (4)	154
C5—H5⋯*Cg*5^ii^	0.95	3.03	3.759 (4)	135

Symmetry codes: (i) 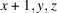 ; (ii) 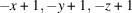 .

**Table 2:** Experimental details

Crystal data	
Chemical formula	C_34_H_32_OS_2_
*M*_r_	520.71
Crystal system, space group	Orthorhombic, *P* *b* *c* *a*
Temperature (K)	168
*a*, *b*, *c* (Å)	11.874 (4), 19.579 (7), 24.374 (9)
*V* (Å^3^)	5666 (3)
*Z*	8
Radiation type	Mo *K*α
μ (mm^−1^)	0.21
Crystal size (mm)	0.68 × 0.40 × 0.13

Data collection	
Diffractometer	Bruker SMART CCD
Absorption correction	Multi-scan (*SADABS*; Krause *et al.*, 2015 ▸)
*T*_min_, *T*_max_	0.835, 1.000
No. of measured, independent and observed [*I* > 2σ(*I*)] reflections	68261, 5679, 3354
*R*_int_	0.134
(sin θ/λ)_max_ (Å^−1^)	0.624

Refinement	
*R*[*F* ^2^ > 2σ(*F* ^2^)], *wR*(*F* ^2^), *S*	0.050, 0.127, 1.06
No. of reflections	5679
No. of parameters	339
H-atom treatment	H-atom parameters constrained
Δρ_max_, Δρ_min_ (e Å^−3^)	0.33, −0.28

Computer programs: *SMART* and *SAINT* (Bruker, 1997 ▸), *SHELXT* (Sheldrick, 2015*a* ▸), *SHELXL2019/2* (Sheldrick, 2015*b* ▸), *Mercury* (Macrae *et al.*, 2020 ▸) and *publCIF* (Westrip 2010 ▸).

## supplementary crystallographic information

### Crystal data

**Table:**

C_34_H_32_OS_2_	*D*_x_ = 1.221 Mg m^−^^3^
*M**_r_* = 520.71	Mo *K*α radiation, λ = 0.71073 Å
Orthorhombic, *P**b**c**a*	Cell parameters from 5483 reflections
*a* = 11.874 (4) Å	θ = 2.2–26.3°
*b* = 19.579 (7) Å	µ = 0.21 mm^−^^1^
*c* = 24.374 (9) Å	*T* = 168 K
*V* = 5666 (3) Å^3^	Plate, pale yellow
*Z* = 8	0.68 × 0.40 × 0.13 mm
*F*(000) = 2208	

### Data collection

**Table:**

Bruker SMART CCD diffractometer	3354 reflections with *I* > 2σ(*I*)
Radiation source: sealed tube	*R*_int_ = 0.134
ω scans	θ_max_ = 26.3°, θ_min_ = 2.2°
Absorption correction: multi-scan (SADABS; Krause *et al.*, 2015)	*h* = −14→14
*T*_min_ = 0.835, *T*_max_ = 1.000	*k* = −11→24
68261 measured reflections	*l* = −30→30
5679 independent reflections	

### Refinement

**Table:**

Refinement on *F*^2^	Primary atom site location: dual
Least-squares matrix: full	Hydrogen site location: inferred from neighbouring sites
*R*[*F*^2^ > 2σ(*F*^2^)] = 0.050	H-atom parameters constrained
*wR*(*F*^2^) = 0.127	*w* = 1/[σ^2^(*F*_o_^2^) + (0.0365*P*)^2^ + 6.2206*P*] where *P* = (*F*_o_^2^ + 2*F*_c_^2^)/3
*S* = 1.06	(Δ/σ)_max_ < 0.001
5679 reflections	Δρ_max_ = 0.33 e Å^−^^3^
339 parameters	Δρ_min_ = −0.28 e Å^−^^3^
0 restraints	

### Special details

**Table:**

Geometry. All esds (except the esd in the dihedral angle between two l.s. planes) are estimated using the full covariance matrix. The cell esds are taken into account individually in the estimation of esds in distances, angles and torsion angles; correlations between esds in cell parameters are only used when they are defined by crystal symmetry. An approximate (isotropic) treatment of cell esds is used for estimating esds involving l.s. planes.

### Fractional atomic coordinates and isotropic or equivalent isotropic displacement parameters (Å^2^)

**Table:**

	*x*	*y*	*z*	*U*_iso_*/*U*_eq_	
S1	0.44124 (7)	0.28091 (4)	0.43352 (3)	0.03261 (19)	
S2	0.43855 (7)	0.41835 (4)	0.49632 (3)	0.03191 (19)	
O2	0.19441 (19)	0.27784 (11)	0.47083 (10)	0.0472 (6)	
H2O	0.239891	0.250347	0.456374	0.071*	
C101	0.5406 (3)	0.38957 (15)	0.38412 (12)	0.0314 (7)	
C102	0.4540 (2)	0.34209 (14)	0.37906 (11)	0.0304 (7)	
C103	0.3799 (3)	0.34092 (15)	0.33207 (12)	0.0316 (7)	
C111	0.2886 (3)	0.28677 (16)	0.32557 (12)	0.0366 (7)	
H111	0.262395	0.273779	0.363137	0.044*	
C112	0.3394 (3)	0.22282 (18)	0.29887 (17)	0.0614 (11)	
H11D	0.280968	0.187848	0.294893	0.092*	
H11E	0.400322	0.205180	0.322025	0.092*	
H11F	0.369497	0.234602	0.262637	0.092*	
C113	0.1849 (3)	0.3108 (2)	0.29280 (15)	0.0574 (10)	
H11A	0.125411	0.276258	0.295201	0.086*	
H11B	0.205896	0.317552	0.254296	0.086*	
H11C	0.157338	0.354035	0.308078	0.086*	
C104	0.3978 (3)	0.38892 (15)	0.29172 (12)	0.0363 (8)	
H104	0.350863	0.388457	0.260141	0.044*	
C105	0.4996 (3)	0.48813 (17)	0.25314 (14)	0.0503 (10)	
H105	0.452517	0.487305	0.221655	0.060*	
C106	0.5824 (4)	0.53643 (19)	0.25746 (15)	0.0609 (11)	
H106	0.593005	0.568410	0.228608	0.073*	
C107	0.6523 (3)	0.53936 (18)	0.30425 (14)	0.0528 (10)	
H107	0.708449	0.573768	0.306929	0.063*	
C108	0.6396 (3)	0.49259 (16)	0.34607 (13)	0.0414 (8)	
H108	0.686723	0.495194	0.377498	0.050*	
C109	0.5563 (3)	0.44036 (15)	0.34254 (12)	0.0336 (7)	
C110	0.4837 (3)	0.43908 (16)	0.29560 (12)	0.0370 (8)	
C201	0.6210 (3)	0.38596 (14)	0.43239 (12)	0.0300 (7)	
C202	0.5861 (2)	0.40046 (14)	0.48581 (12)	0.0303 (7)	
C203	0.6628 (3)	0.40156 (14)	0.53151 (12)	0.0306 (7)	
C211	0.6236 (3)	0.42082 (15)	0.58962 (12)	0.0354 (7)	
H211	0.561121	0.454716	0.585743	0.042*	
C212	0.5756 (3)	0.35802 (17)	0.61924 (13)	0.0485 (9)	
H21A	0.551202	0.371053	0.656198	0.073*	
H21B	0.633787	0.322683	0.621808	0.073*	
H21C	0.511081	0.340249	0.598607	0.073*	
C213	0.7165 (3)	0.45444 (18)	0.62422 (14)	0.0519 (9)	
H21D	0.746040	0.494416	0.604760	0.078*	
H21E	0.777538	0.421601	0.630341	0.078*	
H21F	0.685264	0.468733	0.659612	0.078*	
C204	0.7736 (3)	0.38353 (14)	0.52109 (12)	0.0324 (7)	
H204	0.826086	0.384480	0.550514	0.039*	
C205	0.9258 (3)	0.34162 (15)	0.45912 (13)	0.0362 (7)	
H205	0.976911	0.340143	0.489093	0.043*	
C206	0.9616 (3)	0.32265 (16)	0.40766 (14)	0.0435 (8)	
H206	1.037199	0.308329	0.402203	0.052*	
C207	0.8859 (3)	0.32443 (18)	0.36282 (14)	0.0464 (9)	
H207	0.911225	0.311034	0.327445	0.056*	
C208	0.7764 (3)	0.34529 (16)	0.36978 (13)	0.0411 (8)	
H208	0.726915	0.346307	0.339167	0.049*	
C209	0.7360 (3)	0.36552 (14)	0.42274 (12)	0.0314 (7)	
C210	0.8123 (3)	0.36355 (14)	0.46790 (12)	0.0323 (7)	
C1	0.3767 (3)	0.33266 (15)	0.48761 (12)	0.0325 (7)	
H1	0.387627	0.307626	0.522978	0.039*	
C2	0.2491 (3)	0.34195 (15)	0.47961 (12)	0.0344 (7)	
H2	0.236419	0.371453	0.446697	0.041*	
C3	0.1923 (3)	0.37540 (16)	0.52906 (13)	0.0373 (8)	
C4	0.1482 (3)	0.44076 (17)	0.52523 (16)	0.0495 (9)	
H4	0.159453	0.466572	0.492683	0.059*	
C5	0.0874 (3)	0.4691 (2)	0.5686 (2)	0.0727 (14)	
H5	0.058165	0.514127	0.565737	0.087*	
C6	0.0700 (4)	0.4318 (3)	0.6153 (2)	0.0806 (16)	
H6	0.026471	0.450263	0.644453	0.097*	
C7	0.1160 (4)	0.3668 (3)	0.62003 (17)	0.0747 (14)	
H7	0.105793	0.341571	0.652939	0.090*	
C8	0.1771 (3)	0.3385 (2)	0.57685 (14)	0.0536 (10)	
H8	0.208080	0.293923	0.580218	0.064*	

### Atomic displacement parameters (Å^2^)

**Table:**

	*U*^11^	*U*^22^	*U*^33^	*U*^12^	*U*^13^	*U*^23^
S1	0.0432 (5)	0.0266 (4)	0.0280 (4)	0.0020 (4)	0.0017 (4)	0.0016 (3)
S2	0.0340 (4)	0.0287 (4)	0.0330 (4)	0.0016 (3)	0.0011 (4)	−0.0015 (3)
O2	0.0445 (14)	0.0360 (12)	0.0610 (16)	−0.0023 (11)	0.0005 (12)	−0.0121 (12)
C101	0.0372 (18)	0.0312 (16)	0.0258 (16)	0.0047 (14)	0.0016 (13)	0.0002 (12)
C102	0.0374 (18)	0.0290 (15)	0.0248 (16)	0.0053 (14)	0.0024 (14)	−0.0006 (12)
C103	0.0364 (18)	0.0324 (16)	0.0259 (16)	0.0013 (14)	0.0006 (14)	−0.0007 (13)
C111	0.0411 (19)	0.0415 (18)	0.0272 (16)	−0.0055 (15)	−0.0023 (14)	−0.0005 (14)
C112	0.066 (3)	0.047 (2)	0.071 (3)	−0.013 (2)	0.008 (2)	−0.018 (2)
C113	0.046 (2)	0.072 (3)	0.054 (2)	−0.014 (2)	−0.0098 (19)	0.009 (2)
C104	0.0447 (19)	0.0411 (18)	0.0230 (16)	−0.0018 (15)	−0.0044 (14)	0.0012 (14)
C105	0.069 (3)	0.051 (2)	0.0312 (18)	−0.015 (2)	−0.0113 (18)	0.0129 (16)
C106	0.085 (3)	0.058 (2)	0.040 (2)	−0.023 (2)	−0.004 (2)	0.0179 (18)
C107	0.061 (2)	0.053 (2)	0.045 (2)	−0.0199 (19)	0.0012 (19)	0.0107 (18)
C108	0.046 (2)	0.0446 (19)	0.0338 (18)	−0.0063 (16)	0.0009 (16)	−0.0003 (15)
C109	0.0391 (18)	0.0336 (16)	0.0281 (16)	−0.0001 (15)	0.0027 (15)	0.0000 (13)
C110	0.0453 (19)	0.0389 (18)	0.0268 (17)	−0.0035 (15)	0.0006 (15)	0.0024 (14)
C201	0.0350 (17)	0.0278 (15)	0.0271 (16)	−0.0034 (13)	−0.0043 (14)	0.0041 (13)
C202	0.0347 (18)	0.0243 (15)	0.0318 (17)	0.0003 (12)	−0.0004 (13)	0.0017 (12)
C203	0.0399 (19)	0.0225 (14)	0.0295 (16)	−0.0013 (13)	−0.0045 (14)	0.0007 (12)
C211	0.0448 (19)	0.0318 (16)	0.0295 (17)	0.0034 (15)	−0.0044 (15)	−0.0026 (13)
C212	0.066 (2)	0.046 (2)	0.0333 (19)	0.0007 (18)	0.0034 (18)	0.0020 (15)
C213	0.059 (2)	0.058 (2)	0.038 (2)	−0.0046 (19)	−0.0031 (18)	−0.0152 (17)
C204	0.0395 (19)	0.0291 (16)	0.0287 (16)	−0.0030 (14)	−0.0087 (14)	0.0020 (13)
C205	0.0345 (18)	0.0357 (17)	0.0386 (18)	−0.0013 (15)	−0.0025 (15)	0.0046 (14)
C206	0.036 (2)	0.0420 (19)	0.052 (2)	0.0065 (15)	0.0046 (17)	0.0021 (16)
C207	0.044 (2)	0.058 (2)	0.0363 (19)	0.0071 (18)	0.0030 (17)	−0.0044 (17)
C208	0.041 (2)	0.049 (2)	0.0329 (18)	0.0042 (16)	−0.0042 (15)	−0.0043 (15)
C209	0.0342 (18)	0.0299 (16)	0.0301 (17)	−0.0004 (13)	0.0011 (14)	−0.0009 (13)
C210	0.0349 (18)	0.0262 (16)	0.0358 (18)	−0.0011 (13)	−0.0020 (14)	0.0012 (13)
C1	0.0381 (18)	0.0311 (16)	0.0282 (16)	−0.0001 (14)	0.0030 (14)	−0.0022 (13)
C2	0.0397 (18)	0.0275 (16)	0.0361 (18)	0.0000 (14)	−0.0024 (14)	−0.0016 (13)
C3	0.0303 (17)	0.0368 (18)	0.045 (2)	−0.0073 (14)	0.0023 (15)	−0.0087 (15)
C4	0.041 (2)	0.0393 (19)	0.068 (3)	−0.0021 (16)	0.0074 (18)	−0.0106 (18)
C5	0.047 (3)	0.057 (3)	0.114 (4)	−0.009 (2)	0.023 (3)	−0.046 (3)
C6	0.063 (3)	0.099 (4)	0.080 (3)	−0.041 (3)	0.036 (3)	−0.059 (3)
C7	0.080 (3)	0.096 (4)	0.049 (2)	−0.049 (3)	0.016 (2)	−0.017 (2)
C8	0.058 (2)	0.059 (2)	0.044 (2)	−0.018 (2)	0.0047 (19)	−0.0076 (18)

### Geometric parameters (Å, º)

**Table:**

S1—C102	1.794 (3)	C203—C211	1.538 (4)
S1—C1	1.831 (3)	C211—C212	1.536 (4)
S2—C202	1.805 (3)	C211—C213	1.537 (4)
S2—C1	1.844 (3)	C211—H211	1.0000
O2—C2	1.429 (3)	C212—H21A	0.9800
O2—H2O	0.8400	C212—H21B	0.9800
C101—C102	1.391 (4)	C212—H21C	0.9800
C101—C109	1.432 (4)	C213—H21D	0.9800
C101—C201	1.517 (4)	C213—H21E	0.9800
C102—C103	1.444 (4)	C213—H21F	0.9800
C103—C104	1.377 (4)	C204—C210	1.430 (4)
C103—C111	1.525 (4)	C204—H204	0.9500
C111—C112	1.535 (4)	C205—C206	1.375 (4)
C111—C113	1.542 (4)	C205—C210	1.430 (4)
C111—H111	1.0000	C205—H205	0.9500
C112—H11D	0.9800	C206—C207	1.415 (5)
C112—H11E	0.9800	C206—H206	0.9500
C112—H11F	0.9800	C207—C208	1.374 (4)
C113—H11A	0.9800	C207—H207	0.9500
C113—H11B	0.9800	C208—C209	1.433 (4)
C113—H11C	0.9800	C208—H208	0.9500
C104—C110	1.420 (4)	C209—C210	1.426 (4)
C104—H104	0.9500	C1—C2	1.538 (4)
C105—C106	1.368 (5)	C1—H1	1.0000
C105—C110	1.425 (4)	C2—C3	1.529 (4)
C105—H105	0.9500	C2—H2	1.0000
C106—C107	1.411 (5)	C3—C8	1.383 (5)
C106—H106	0.9500	C3—C4	1.386 (4)
C107—C108	1.379 (4)	C4—C5	1.395 (5)
C107—H107	0.9500	C4—H4	0.9500
C108—C109	1.425 (4)	C5—C6	1.368 (6)
C108—H108	0.9500	C5—H5	0.9500
C109—C110	1.432 (4)	C6—C7	1.389 (7)
C201—C202	1.396 (4)	C6—H6	0.9500
C201—C209	1.442 (4)	C7—C8	1.394 (5)
C202—C203	1.439 (4)	C7—H7	0.9500
C203—C204	1.386 (4)	C8—H8	0.9500
			
C102—S1—C1	101.47 (13)	C203—C211—H211	107.5
C202—S2—C1	101.17 (13)	C211—C212—H21A	109.5
C2—O2—H2O	109.5	C211—C212—H21B	109.5
C102—C101—C109	119.8 (3)	H21A—C212—H21B	109.5
C102—C101—C201	120.2 (3)	C211—C212—H21C	109.5
C109—C101—C201	119.9 (3)	H21A—C212—H21C	109.5
C101—C102—C103	122.1 (3)	H21B—C212—H21C	109.5
C101—C102—S1	116.3 (2)	C211—C213—H21D	109.5
C103—C102—S1	121.6 (2)	C211—C213—H21E	109.5
C104—C103—C102	117.5 (3)	H21D—C213—H21E	109.5
C104—C103—C111	120.6 (3)	C211—C213—H21F	109.5
C102—C103—C111	121.8 (3)	H21D—C213—H21F	109.5
C103—C111—C112	109.4 (3)	H21E—C213—H21F	109.5
C103—C111—C113	114.2 (3)	C203—C204—C210	122.7 (3)
C112—C111—C113	110.1 (3)	C203—C204—H204	118.6
C103—C111—H111	107.6	C210—C204—H204	118.6
C112—C111—H111	107.6	C206—C205—C210	120.6 (3)
C113—C111—H111	107.6	C206—C205—H205	119.7
C111—C112—H11D	109.5	C210—C205—H205	119.7
C111—C112—H11E	109.5	C205—C206—C207	120.1 (3)
H11D—C112—H11E	109.5	C205—C206—H206	119.9
C111—C112—H11F	109.5	C207—C206—H206	119.9
H11D—C112—H11F	109.5	C208—C207—C206	120.9 (3)
H11E—C112—H11F	109.5	C208—C207—H207	119.6
C111—C113—H11A	109.5	C206—C207—H207	119.6
C111—C113—H11B	109.5	C207—C208—C209	120.7 (3)
H11A—C113—H11B	109.5	C207—C208—H208	119.7
C111—C113—H11C	109.5	C209—C208—H208	119.7
H11A—C113—H11C	109.5	C210—C209—C208	118.4 (3)
H11B—C113—H11C	109.5	C210—C209—C201	118.9 (3)
C103—C104—C110	122.3 (3)	C208—C209—C201	122.7 (3)
C103—C104—H104	118.8	C209—C210—C204	119.2 (3)
C110—C104—H104	118.8	C209—C210—C205	119.4 (3)
C106—C105—C110	120.4 (3)	C204—C210—C205	121.4 (3)
C106—C105—H105	119.8	C2—C1—S1	112.7 (2)
C110—C105—H105	119.8	C2—C1—S2	107.4 (2)
C105—C106—C107	120.8 (3)	S1—C1—S2	114.81 (16)
C105—C106—H106	119.6	C2—C1—H1	107.2
C107—C106—H106	119.6	S1—C1—H1	107.2
C108—C107—C106	120.4 (3)	S2—C1—H1	107.2
C108—C107—H107	119.8	O2—C2—C3	107.1 (2)
C106—C107—H107	119.8	O2—C2—C1	111.3 (2)
C107—C108—C109	120.5 (3)	C3—C2—C1	112.7 (3)
C107—C108—H108	119.7	O2—C2—H2	108.6
C109—C108—H108	119.7	C3—C2—H2	108.6
C108—C109—C101	123.1 (3)	C1—C2—H2	108.6
C108—C109—C110	118.6 (3)	C8—C3—C4	119.4 (3)
C101—C109—C110	118.3 (3)	C8—C3—C2	119.8 (3)
C104—C110—C105	120.9 (3)	C4—C3—C2	120.6 (3)
C104—C110—C109	119.9 (3)	C3—C4—C5	120.8 (4)
C105—C110—C109	119.2 (3)	C3—C4—H4	119.6
C202—C201—C209	119.3 (3)	C5—C4—H4	119.6
C202—C201—C101	121.8 (3)	C6—C5—C4	119.7 (4)
C209—C201—C101	118.9 (3)	C6—C5—H5	120.1
C201—C202—C203	122.5 (3)	C4—C5—H5	120.1
C201—C202—S2	117.4 (2)	C5—C6—C7	119.9 (4)
C203—C202—S2	120.1 (2)	C5—C6—H6	120.0
C204—C203—C202	117.1 (3)	C7—C6—H6	120.0
C204—C203—C211	121.2 (3)	C6—C7—C8	120.5 (4)
C202—C203—C211	121.6 (3)	C6—C7—H7	119.8
C212—C211—C213	110.6 (3)	C8—C7—H7	119.8
C212—C211—C203	110.4 (2)	C3—C8—C7	119.7 (4)
C213—C211—C203	113.1 (3)	C3—C8—H8	120.1
C212—C211—H211	107.5	C7—C8—H8	120.1
C213—C211—H211	107.5		
			
C109—C101—C102—C103	1.6 (4)	C201—C202—C203—C211	177.2 (3)
C201—C101—C102—C103	−176.0 (3)	S2—C202—C203—C211	−2.7 (4)
C109—C101—C102—S1	−179.7 (2)	C204—C203—C211—C212	−93.8 (3)
C201—C101—C102—S1	2.6 (4)	C202—C203—C211—C212	85.2 (3)
C1—S1—C102—C101	76.0 (2)	C204—C203—C211—C213	30.7 (4)
C1—S1—C102—C103	−105.3 (2)	C202—C203—C211—C213	−150.2 (3)
C101—C102—C103—C104	0.1 (4)	C202—C203—C204—C210	−0.9 (4)
S1—C102—C103—C104	−178.5 (2)	C211—C203—C204—C210	178.2 (3)
C101—C102—C103—C111	177.2 (3)	C210—C205—C206—C207	−0.2 (5)
S1—C102—C103—C111	−1.4 (4)	C205—C206—C207—C208	0.3 (5)
C104—C103—C111—C112	91.7 (4)	C206—C207—C208—C209	−0.2 (5)
C102—C103—C111—C112	−85.3 (4)	C207—C208—C209—C210	0.1 (5)
C104—C103—C111—C113	−32.2 (4)	C207—C208—C209—C201	−178.2 (3)
C102—C103—C111—C113	150.8 (3)	C202—C201—C209—C210	−2.7 (4)
C102—C103—C104—C110	−1.2 (4)	C101—C201—C209—C210	178.6 (3)
C111—C103—C104—C110	−178.4 (3)	C202—C201—C209—C208	175.6 (3)
C110—C105—C106—C107	−1.0 (6)	C101—C201—C209—C208	−3.0 (4)
C105—C106—C107—C108	1.2 (6)	C208—C209—C210—C204	−180.0 (3)
C106—C107—C108—C109	0.4 (5)	C201—C209—C210—C204	−1.6 (4)
C107—C108—C109—C101	178.7 (3)	C208—C209—C210—C205	0.0 (4)
C107—C108—C109—C110	−2.2 (5)	C201—C209—C210—C205	178.4 (3)
C102—C101—C109—C108	177.0 (3)	C203—C204—C210—C209	3.4 (4)
C201—C101—C109—C108	−5.4 (4)	C203—C204—C210—C205	−176.5 (3)
C102—C101—C109—C110	−2.1 (4)	C206—C205—C210—C209	0.1 (4)
C201—C101—C109—C110	175.5 (3)	C206—C205—C210—C204	−179.9 (3)
C103—C104—C110—C105	−179.8 (3)	C102—S1—C1—C2	79.4 (2)
C103—C104—C110—C109	0.7 (5)	C102—S1—C1—S2	−44.0 (2)
C106—C105—C110—C104	179.7 (3)	C202—S2—C1—C2	−164.7 (2)
C106—C105—C110—C109	−0.8 (5)	C202—S2—C1—S1	−38.5 (2)
C108—C109—C110—C104	−178.1 (3)	S1—C1—C2—O2	50.8 (3)
C101—C109—C110—C104	1.0 (4)	S2—C1—C2—O2	178.23 (19)
C108—C109—C110—C105	2.4 (5)	S1—C1—C2—C3	171.0 (2)
C101—C109—C110—C105	−178.5 (3)	S2—C1—C2—C3	−61.5 (3)
C102—C101—C201—C202	−68.8 (4)	O2—C2—C3—C8	49.5 (4)
C109—C101—C201—C202	113.6 (3)	C1—C2—C3—C8	−73.2 (4)
C102—C101—C201—C209	109.8 (3)	O2—C2—C3—C4	−125.6 (3)
C109—C101—C201—C209	−67.8 (4)	C1—C2—C3—C4	111.7 (3)
C209—C201—C202—C203	5.5 (4)	C8—C3—C4—C5	−1.0 (5)
C101—C201—C202—C203	−175.9 (3)	C2—C3—C4—C5	174.2 (3)
C209—C201—C202—S2	−174.6 (2)	C3—C4—C5—C6	−0.7 (6)
C101—C201—C202—S2	4.0 (4)	C4—C5—C6—C7	2.1 (6)
C1—S2—C202—C201	72.4 (2)	C5—C6—C7—C8	−2.0 (6)
C1—S2—C202—C203	−107.7 (2)	C4—C3—C8—C7	1.1 (5)
C201—C202—C203—C204	−3.7 (4)	C2—C3—C8—C7	−174.0 (3)
S2—C202—C203—C204	176.4 (2)	C6—C7—C8—C3	0.3 (6)

### Hydrogen-bond geometry (Å, º)

*Cg*1 and *Cg*5 are the centroids of the C3–C8 and C205–C210 rings, respectively.

**Table:**

*D*—H···*A*	*D*—H	H···*A*	*D*···*A*	*D*—H···*A*
O2—H2*O*···S1	0.84	2.53	3.069 (3)	123
C111—H111···S1	1.00	2.73	3.197 (3)	109
C211—H211···S2	1.00	2.72	3.163 (3)	107
C206—H206···O2^i^	0.95	2.58	3.284 (4)	132
C205—H205···*Cg*1^i^	0.95	3.01	3.886 (4)	154
C5—H5···*Cg*5^ii^	0.95	3.03	3.759 (4)	135

Symmetry codes: (i) *x*+1, *y*, *z*; (ii) −*x*+1, −*y*+1, −*z*+1.
